# Pulmonary Remodeling in Equine Asthma: What Do We Know about Mediators of Inflammation in the Horse?

**DOI:** 10.1155/2016/5693205

**Published:** 2016-12-07

**Authors:** Ann Kristin Barton, Heidrun Gehlen

**Affiliations:** Equine Clinic, Freie Universitaet Berlin, Berlin, Germany

## Abstract

Equine inflammatory airway disease (IAD) and recurrent airway obstruction (RAO) represent a spectrum of chronic inflammatory disease of the airways in horses resembling human asthma in many aspects. Therefore, both are now described as severity grades of equine asthma. Increasing evidence in horses and humans suggests that local pulmonary inflammation is influenced by systemic inflammatory processes and the other way around. Inflammation, coagulation, and fibrinolysis as well as extracellular remodeling show close interactions. Cytology of bronchoalveolar lavage fluid and tracheal wash is commonly used to evaluate the severity of local inflammation in the lung. Other mediators of inflammation, like interleukins involved in the chemotaxis of neutrophils, have been studied. Chronic obstructive pneumopathies lead to remodeling of bronchial walls and lung parenchyma, ultimately causing fibrosis. Matrix metalloproteinases (MMPs) are discussed as the most important proteolytic enzymes during remodeling in human medicine and increasing evidence exists for the horse as well. A systemic involvement has been shown for severe equine asthma by increased acute phase proteins like serum amyloid A and haptoglobin in peripheral blood during exacerbation. Studies focusing on these and further possible inflammatory markers for chronic respiratory disease in the horse are discussed in this review of the literature.

## 1. Introduction

Disorders of the respiratory system, particularly the lower airways, are the most frequently diagnosed conditions in sport horses evaluated for poor performance [1, 2]. Equine recurrent airway obstruction (RAO) and IAD represent a spectrum of chronic inflammatory disease of the airways in horses resembling human asthma in many aspects [3–5]. Therefore, the term equine asthma has been suggested to include inflammatory airway disease (IAD) as mild-to-moderate and recurrent airway obstruction (RAO) as severe equine asthma [5]. Parallels of human and equine disease are shown in Table 1. RAO and IAD are characterized by the absence of signs of acute infection like fever or leukocytosis, duration of more than 4 weeks, neutrophilic inflammation in bronchial secretion, airway hyperresponsiveness, and subclinical versus reversible airway obstruction [3, 6]. While human asthma has been commonly described as an eosinophilic disease, it is lately more and more recognized that neutrophilic inflammation may be present in human asthma of all severities as well, in particular in severe asthmatic patients and during acute disease exacerbation [7–9]. On the other hand, eosinophils, metachromatic cells, or neutrophils characterize the subforms of mild-to-moderate equine asthma or IAD [3]. IAD often occurs coincidentally with exercise-induced pulmonary hemorrhage (EIPH) in racehorses [10–13]. The later does not have an allergic background, but inflammation following stress failures of pulmonary capillaries may be a trigger factor for the development of IAD in these subjects. IAD and EIPH also play a role in warmbloods; nevertheless, the most common lower airway disease in these horses is RAO. The estimated prevalence in the northern hemisphere is about 14% [6] with incidence and severity of the disease increasing with age and stabling, so that RAO is a common reason for the career's end of sport horses [14]. Due to the fact that RAO was much better defined over the last 20 years than IAD, literature on equine asthma mainly focused on RAO. Although an increased risk for IAD horses to develop RAO has been described, most horses seem to recover. Therefore it is essential not to use equine asthma as a general term for both diseases but to be aware of the differentiation between mild-to- moderate (IAD) and severe equine asthma (RAO).

Since bronchoalveolar lavage (BAL) using fiber-optic endoscopy was first described in horses [15], cytological and microbiological evaluation of tracheal washes (TW) and BAL fluid (BALF) have become the cornerstones in the diagnosis of respiratory disease alongside clinical and functional examinations. Although severely asthmatic horses often show easily visible signs of disease, difficulties may arise in clinical practice due to the fact that patients may be presented in remission. Mild-to-moderate equine asthma tends to occur subclinically as well. A natural challenge test including exposure of horses to mouldy hay and straw is recommended for research purposes to differentiate between mild-to-moderate and severe forms of equine asthma, but not for clinical routine [3].

In these cases, commonly used examination techniques may be insufficient for diagnosis and evaluation of treatment success. Therefore, multiple studies have been performed to establish further inflammatory markers for equine respiratory disease. Evidence exists that systemic involvement may exist in severe equine asthma as has been shown for human asthma [16] and chronic obstructive pulmonary disease (COPD) [17, 18]. Therefore, not only markers for pulmonary inflammation dominated by neutrophils, but also systemic markers in peripheral blood may be rewarding in the evaluation of equine disease.

## 2. Local Inflammatory Markers in TW and BALF

Neutrophilia in TW and BALF is a predominant cytological feature of inflammation in mild-to-moderate and in particular in severe equine asthma [19–22], in which neutrophils migrate within hours into the airway lumen, followed by the development of airway obstruction and a late phase of migration [22–24]. Neutrophil recruitment to the airway lumen also occurs in acute asthma exacerbations as early as 4 hours after allergen challenge [25–27]. Reference values for BALF cytology in healthy include a total nucleated cell count ≤ 530 cells/lL, neutrophils ≤ 5%, eosinophils ≤ 1%, and metachromatic cells ≤ 2% based on a 250 mL infusion volume [3]. In exacerbations of severe equine asthma or RAO horses present with dyspnea at rest, as shown by a maximum interpleural pressure > 15 cmH_2_O caused by bronchoconstriction, mucosal swelling and mucus accumulation [6], and inflammation of the small airways, in which neutrophils exceed 25% in BALF cytology [28]. The definitive diagnosis of IAD is also based on BALF cytology [3], which is characterized by an increase in the total nucleated cell count with mild neutrophilia [29–32] and lymphocytosis [31, 33, 34] or, alternatively, by increased mast cell [35, 36] or eosinophil counts [37].

The immunologic background of severe equine asthma remains not fully elucidated despite many studies on the pathogenesis [38–41] and the therapeutic approach to exacerbation [42–53]. Although evidence for a Th-2 based hypersensitivity reaction of allergy type IV tends to overwhelm [54–56], there is also evidence for a Th-1 based immunologic background of the disease. Increased levels of interleukins 4 and 5 as well as decreased Interferon-*γ* expression support a Th-2 base [56]. Lavoie et al. [54] showed that recombinant equine IL-4 causes morphological changes in blood neutrophils, increases IL-8 mRNA levels, and potentiates effects of LPS and TNF-*α* on IL-8 expression by pulmonary artery endothelial cells. Receptors for IL-4, however, were not increased on neutrophils in severe equine asthma compared to healthy horses, but a genetic linkage of polymorphisms in IL-4-receptor-*α* on chromosome 13 with severe equine asthma was found and increased expression of this receptor in one high-prevalence family of horses, but not in another one [57]. On the other hand, Ainsworth et al. [38] demonstrated increased interferon-*γ* production in BAL cells, which supports a Th1-based background for RAO, while still others suggest no involvement of either type response in this disease [58]. Inhalation of immune modulatory, specific bacterial DNA-sequences (CpG-nanoparticles) modified the profile of expressed cytokines in RAO patients towards a Th-1 profile and was accompanied by a marked reduction in clinical signs and neutrophilia in TW [59]. IL-8 is the predominant chemokine for neutrophils and was shown to be increased in BALF within hours after changing roughage feed from grass silage to hay in RAO affected horses [60]. In chronic inflammation IL-17 [61] and IL-1*β* and IL-23 also play a role [62].

While the percentage of neutrophils in BALF decreases to reference levels in phases of remission, which hinders cytological diagnosis of the disease, myeloperoxidase (MPO) concentration in BALF was significantly higher in severe equine asthma during either crisis or remission compared to control horses [63]. Therefore, MPO may serve as a sensitive inflammatory marker in subclinical cases [64]. In unison to this, several studies demonstrated reduced antioxidative capacity in severe equine asthma as evidenced by low ascorbic acid concentrations in BALF [65–69]. During recovery from exacerbation, the reduction in ascorbic acid is followed by an increase in BALF glutathione peroxidase activity, presumably to combat disease-associated oxidative stress [65, 66]. Furthermore, markers of oxidative stress have been identified within the airways and in exhaled breath condensate in severe equine asthma, but not following short-term organic dust inhalation challenge [56, 68].

## 3. Systemic Inflammatory Markers in the Blood

Pulmonary neutrophilic inflammation during exacerbation has been the focus of many studies. It is largely reversible by antigen withdrawal in a low-dust environment [6, 28]. However, as in neutrophilic human asthma [70], evidence exists that the inflammatory processes are not completely resolved as residual airway bronchoconstriction, elevated smooth muscle cell turnover surrounding the airways, and higher nuclear factor *κ*B (NF-*κ*B) activity are observable in asymptomatic horses affected with severe equine asthma [4, 71, 72]. NF-*κ*B is a specific transcription factor that has a key role in inflammatory processes. It also plays a role in airway smooth muscle (ASM) cells phenotype modulation, characterized by reversible switching between contractile and proliferative phenotypes, which is considered to contribute to airway proliferation in human asthma [7]. NF-*κ*B has also been reported as a key regulator for the occurrence and development of equine asthma. During disease exacerbation, peripheral blood leucocyte activation and increased concentration of circulatory inflammatory mediators have been observed in affected horses, suggesting that the inflammatory process might not be limited to the lung [73–77]. Human asthma is considered to be a systemic disease, as an increase in several inflammatory markers has been observed in the blood of affected patients [16]. These include immunity-related mediators (cytokines, eicosanoids, cyclooxygenase products, and IgEs) and the acute phase markers C-reactive protein, haptoglobin, fibrinogen, and serum amyloid A [78–80]. Systemic inflammation in patients with chronic airway diseases is thought to contribute to comorbidities [16, 81–84].

Literature on systemic inflammatory processes in equine asthma is rare. Lavoie-Lamoureux et al. [85] compared several acute phase proteins (haptoglobin, serum amyloid A, and C-reactive protein) and cytokines (interleukins 2, 4, and 10 as well as interferon-*α* und interferon-*γ*) in serum of healthy individuals and horses affected by severe equine asthma over 30 days of exposition to hay and straw. Haptoglobin was found to be a suitable marker for both acute and chronic systemic inflammation, whereas high concentrations of serum amyloid A indicated acute inflammation. There was no difference in serum concentrations of the evaluated cytokines between affected horses and controls. In another study though, increased TNF-*α* concentrations were found after ex vivo stimulation with bacterial products in severe equine asthma [86]. Niedźwiedź et al. [87] found increased markers for oxidative stress in peripheral blood during exacerbation compared to controls. Although these systemic markers seem to be attractive, as repeated cytological examinations from bronchoalveolar lavage samples may not be necessary anymore, they might be hard to use in a clinical setting. Due to their low specificity for pulmonary disease, further unrelated and possibly subclinical disease might have to be excluded, leading to a higher diagnostic effort in the end. As bronchoalveolar lavage is an easy-to-perform procedure in a standing sedated horse, markers from this fluid may be of higher value, in particular in mild-to-moderate equine asthma or disease remission.

Since the 1970s a correlation of sepsis and hypocalcemia has gained increasing attention in research. In sepsis, procalcitonin (PCT) is found in high concentration in the peripheral blood and is expressed by many organ tissues [88]. PCT is a precursor of the hormone calcitonin, which regulates calcium metabolism by inhibition of osteoclasts' activity. In healthy subjects, preprocalcitonin (pre-PCT) is produced exclusively in the thyroid gland. PCT plasma concentrations in acute local inflammatory processes in the lung are much lower than in sepsis but allow differentiation between pulmonary diseases in humans [89–91]. For example, PCT was used for differentiation of tuberculosis from other pneumopathies [92]. In chronic pulmonary disease like human asthma, increased PCT concentrations were found as well. Acute exacerbations are often accompanied by bacterial infections of the lower airways. PCT can be used in the decision for or against antibiotic treatment and may help to interpret diffuse thoracic radiographs [93, 94]. In COPD (chronic obstructive pulmonary disease) PCT eases the decision for antibiotics or glucocorticoids [95]. It is also useful for follow-up in COPD [96].

Rieger et al. [97] established an ELISA specific for equine PCT. Clear differences were found in plasma between septic horses and healthy individuals using this and another ELISA [98, 99]. Increases in systemic PCT concentrations were also found in chronic pneumopathies compared to horses free of respiratory disease in BALF and a trend was also visible in plasma, although this needs to be confirmed in a higher number of samples [100].

## 4. Coagulation and Fibrinolysis

In the long term, chronic obstructive pneumopathies lead to chronic remodeling of bronchial walls and lung parenchyma, which is accompanied by fibrosis formation [101–104]. This remodeling is favored by procoagulatory conditions, while fibrinolysis serves to remove alveolar fibrin deposits and counteracts coagulation and beginning fibrosis [105].

After mechanical and inflammatory defects, which destroy the capillary endothelium and in particular the alveolar epithelium, plasma proteins transudate into the alveolar space and activate coagulation, of which fibrin is the end product. This is part of the natural healing and repair process and serves as a primary occlusion of the membranous defect [106]. On the other hand, excessive and persistent coagulation is pathologic and leads to fibrin deposition and fibrosis formation, which has a negative impact on pathogenesis and progression of multiple respiratory diseases [107, 108].

Apart from favoring fibrosis formation, fibrin and its derivates influence pathomechanisms of inflammation and the further course of the disease and repair of affected lung tissue [109]. Fibrin stimulates migration of inflammatory cells [110, 111] as well as adhesion and proliferation of fibroblasts with following collagen production [112, 113]. The fibrin molecule modulates the inflammatory response by binding to monocytes and activation of transcription factors like NF-*κ*B, which increases proinflammatory cytokine production [114]. In addition, fibrin and its derivates inhibit surfactant, decreasing the alveolar surface tension, favoring micro atelectases, and decreasing gas exchange. This dysfunction is caused by the inclusion of surfactant components into the fibrin matrix and a following dysfunction of the main phospholipid DPPC (Dipalmitoylphosphatidylcholine) [115]. Interactions of surfactant with fibrinogen, fibrin monomers, and other proteins have also been demonstrated; of these fibrin monomers have the strongest inhibitory effect [105].

It has been hypothesized early in human medicine that respiratory disease damaging the alveolar membrane must cause a misbalance between coagulatory and fibrinolytic activity in the extravascular space. Studies on different parameters of fibrinolysis like u-PA (urokinase-type plasminogen activator), PAI-1 (plasminogen activator inhibitor 1), and *α*_2_-AP (alpha 2-antiplasmin) and on d-dimers (a fibrin-degradation product containing two D fragments of the fibrin protein joined by a cross-link) have demonstrated an increase in coagulation in bronchoalveolar lavage fluid (BALF) in patients suffering from chronic respiratory disease [106]. Coagulation and fibrinolysis have been shown to play an important role in the pathomechanisms of numerous pulmonary diseases in humans, and the development of therapeutics supporting fibrinolysis is discussed as a promising approach [116].

Increased coagulatory activity also influences airway smooth muscle (ASM) proliferation. During asthma exacerbation, plasma circulating coagulant factor X (FX) enters the inflamed airways and is activated (FXa). FXa, but not FX, stimulated increases in ASM IL-6 production and cell number [117].

In veterinary medicine and in particular in the equine lung, literature in this field is rare so far. Nevertheless, thickened alveolar interstitial spaces by the accumulation of fibrin and fibrinogen have been found in horses affected by chronic pulmonary disease [118]. In severe equine asthma, increased concentrations of fibrinogen derivates, proteases, and procoagulatory activity could be demonstrated in respiratory secretions. The results of this study allow the assumption that a misbalance of extravascular homeostasis is also a feature of equine asthma and that fibrin deposition, following fibrosis and surfactant dysfunction, supports the progression of the disease. Fibrinolysis has been studied in more detail in other organ systems, for example, in plasma and peritoneal fluid of horses suffering from colic [119, 120] and in synovial fluid of foals affected by arthritis [121].

## 5. Extracellular Remodeling

Remodeling of the extracellular matrix (ECM) of pulmonary connective tissue is a continuous process allowing growth and regeneration. To maintain the tissue's stability, a balance between degradation, in which matrix metalloproteinases are the most important proteolytic enzymes, and resynthesis of extracellular matrix structures is required [122]. The pulmonary interstitium forms the mechanical scaffold of the lung, while the basement membrane supports alveolar epithelial cells and in part determines the resistance of the diffusion barrier [123]. The primary structural fibrils of the lung are composed of type I collagen and elastin. Elastin fibers are very elastic and stable for a long time [124]. The alveolar wall is primarily composed of type III collagen [125], while the basement membrane is rich in type IV collagen. Large collagen and elastin fibers are connected by a variety of smaller fibrils. Degradation of the primary structural fibrils of the lung will therefore involve cleavage of the cross-linking fibrils to expose enzyme binding sites [126]. Multiple enzymes are involved in the turnover of the ECM and it may be impossible to specify a single protease as the critical mediator of any particular pulmonary disease.

The function of matrix metalloproteinases (MMPs) has originally been described over fifty years ago [127]. MMPs play an important role in the turnover of the extracellular matrix (ECM) components, tissue degradation, repair mechanisms, and cell migration [128]. Their best known physiological function is the cleavage and rebuilding of connective tissues [129]. MMPs may play a crucial role in inflammatory reactions by regulating physiologic barriers, modulation of cytokines and chemokines, and establishing a chemokine gradient to regulate the leukocyte accumulation into the inflamed tissue [130].

MMPs are very likely to have a central role in destructive pulmonary disease, characterized by fibrosis formation and the loss of elastin and collagen fibers. Type I collagen is very resistant against enzymatic degradation, which is possible only by few specific MMPs at physiologic pH [131]. Some MMPs are elastases that may also degrade type IV collagen [132]. Several studies have demonstrated a central role of MMPs in chronic respiratory disease in humans and horses. MMPs are assumed to be the major proteolytic enzymes involved in the pathogenesis of COPD [133, 134]. Increased levels of MMP-1 and MMP-9 have been detected in BALF of patients with emphysema, which are produced by macrophages [135, 136].

Several studies have been performed in horses, in which MMPs were evaluated by zymography in BALF samples. In severe equine asthma increased MMP-2 and MMP-9 concentrations were found compared to healthy controls [137]. In particular MMP-9 seems to play a pathophysiologic role [138]. Both MMP-2 and MMP-9 showed a correlation to stable dust concentrations that contains fungal moulds and bacterial endotoxins [139–141]. MMP-9 also correlated to BALF neutrophilia and decreased with neutrophilia after therapy [142]. This was not found for MMP-2, which seems to have a constitutive role in physiologic tissue remodeling [143]. Raulo et al. [144] were also unable to demonstrate increased MMP-2 and MMP-14 activity in TW and BALF in severe equine asthma, but up to 7 times increased activity of MMP-9, MMP-8, and MMP-13, produced by pulmonary macrophages and epithelial cells [144, 145].

It seems desirable to develop therapeutic approaches that protect the lung against overwhelming MMP activity but allow physiologic cell remodeling, which is important in the immunology of healing processes [146]. To prevent uncontrolled turnover of the ECM, inflammation, cellular growth, and migration, MMP activity must be tightly controlled on the levels of transcription, zymogen activity, and by endogenous inhibitors. Overwhelming MMP activity in the horse could be inhibited in vitro with doxycycline [143].

Tissue inhibitors of metalloproteinases (TIMPs) are natural, endogenous MMP-inhibitors that regulate MMP-induced turnover of the ECM, tissue remodeling, and cellular behavior [147, 148]. Next to several MMPs, TIMP-1 and TIMP-2 were found to be increased in tuberculosis in humans [149] and decreased after successful therapy. In COPD, local MMP-9 and TIMP-1 concentrations in BALF were high, while they were low in plasma [150]. In interstitial pneumopathies MMP and TIMP patterns have been discussed as possible prognostic markers and TIMP inhalation as a possible therapeutic approach [151]. There are some synthetic inhibitors that regulate the pathologic effects of MMPs and might support the healing process of epithelial membranes in the airways [147, 148]. TIMP-1 and TIMP-2 levels as well as MMP-TIMP ratios have also been studied in equine chronic pneumopathies [138]. TIMP-1 and TIMP-2 concentrations in BALF were significantly increased in all stages of equine asthma compared to controls. In particular, the MMP-8 : TIMP ratios were found useful to evaluate the severity and character of respiratory disease and may have prognostic value for equine pneumopathies.

## 6. Airway Smooth Muscle Dysfunction

ASM hyperresponsiveness and hyperplasia are a well-known phenomena in severe equine asthma [152]. In vitro and in vivo studies have shown that the proliferative, secretory, and contractile functions of airway smooth muscle (ASM) are dysfunctional in human asthma. The same is true in equine asthma [153], but ASM remodeling is not necessarily associated with pulmonary neutrophilia and clinical status. Leclere et al. [154, 155] found that inhaled corticosteroids may accelerate the reversal of smooth muscle remodeling, even if airway inflammation is better controlled with antigen avoidance. Various mediators are derived from inflammatory cells or produced by ASM itself [156]. It has been shown that ASM is not only an effector of bronchoconstriction, but has additional roles as an immunomodulatory of inflammation and remodeling. ASM can both produce and respond to an array of cytokines, chemokines, and growth factors, leading to cell migration and proliferation, production of ECM proteins, and altered reactivity.

The roles of NF-*κ*ß and FXa have been discussed already. Further key mediators in human asthma include CXC chemokines, Th-17 derived cytokines, and semaphorins [156]. CXCL1, CXCL2, and CXCL3 are expressed by ASM, induce neutrophil chemotaxis, and regulate ASM migration [156–158]. Th17-cells, a subset of T cells distinct from Th1/Th2 cells, produce IL-17 and IL-22. IL-17a contributes to human asthma through recruitment of inflammatory cells including neutrophils, monocytes, and macrophages and by stimulation of the release of MMPs as well as cytokines including IL-6 and IL-8 [159–162]. Disease chronicity of equine asthma has also been associated with Th17-mediated immunity [61, 163]. A trend for upregulation of IL-17 was also found [164]. Th17 cytokines may therefore contribute to the sustained airway neutrophilic inflammation in equine disease, as reported in human asthma [165]. Semaphorins, previously called collapsins, were initially discovered as “axon guidance” cues in neuronal cells [166] but are now known to be ubiquitously expressed. In human asthma, they also contribute to inflammation, hyperresponsiveness, and remodeling by regulating airway angiogenesis, recruitment of fibrocytes, and promotion of myofibroblast hyperplasia [167, 168].

## 7. Conclusions

In conclusion, the results of the studies reviewed here show the complexity in the pathogenesis of respiratory disease in the horse. Many parallels can be drawn to human asthma, but species specific differences have also been found. Therefore, it is a challenge to find new therapeutic approaches to these pathologies with a high economic impact in veterinary medicine. Nevertheless, the numerous inflammatory markers that have been established for horses so far may help to understand the pathogenesis of lower airway disease in more detail and to diagnose the different diseases correctly even in subclinical cases. Further studies should focus on the course of the evaluated markers during therapy to evaluate their use in follow-up after therapy or during long-term therapy and to evaluate the success of not only established, but also new therapeutic approaches for chronic respiratory disease.

## Figures and Tables

**Table 1:** Parallels and differences between equine and humane asthma. IAD = inflammatory airway disease, RAO = recurrent airway obstruction, ASM = airway smooth muscle, and ECM = extracellular matrix.

	Mild-to-moderate equine asthma (IAD)	Severe equine asthma (RAO)	Mild human asthma	Severe human asthma
Airway hyperresponsiveness	Yes	Yes	Yes	Yes
Airway obstruction	Subclinical	Partly reversible	Reversible	Partly reversible
Environmental origin	Yes	Yes	Yes	Yes
Genetic origin	No data	Yes	Yes	Conflicting evidence
ASM dysfunction	No data	Yes	Yes	Yes
ECM remodeling	Conflicting evidence	Yes	Yes	Yes
Airway neutrophilia	Neutrophilic subtype	Yes	Neutrophilic endotype	Yes
Airway eosinophilia	Eosinophilic subtype	No	Yes	Yes

## References

[1] Allen K. J., Tremaine W. H., Franklin S. H. (2006). Prevalence of inflammatory airway disease in national hunt horses referred for investigation of poor athletic performance. *Equine Veterinary Journal. Supplement*.

[2] Martin B. B., Reef V. B., Parente E. J., Sage A. D. (2000). Causes of poor performance of horses during training, racing, or showing: 348 cases (1992–1996). *Journal of the American Veterinary Medical Association*.

[3] Couëtil L. L., Cardwell J. M., Gerber V. (2016). Inflammatory airway disease of horses—revised consensus statement. *Journal of Veterinary Internal Medicine*.

[4] Leclere M., Lavoie-Lamoureux A., Lavoie J.-P. (2011). Heaves, an asthma-like disease of horses. *Respirology*.

[5] Bullone M., Lavoie J.-P. (2015). Asthma ‘of horses and men’—how can equine heaves help us better understand human asthma immunopathology and its functional consequences?. *Molecular Immunology*.

[6] Pirie R. S. (2014). Recurrent airway obstruction: a review. *Equine Veterinary Journal*.

[7] Qiu C., Zhang J., Su M. (2015). Nuclear factor-*κ*B mediates the phenotype switching of airway smooth muscle cells in a murine asthma model. *International Journal of Clinical and Experimental Pathology*.

[8] Nakagome K., Matsushita S., Nagata M. (2012). Neutrophilic inflammation in severe asthma. *International Archives of Allergy and Immunology*.

[9] Wenzel S. E. (2012). Asthma phenotypes: the evolution from clinical to molecular approaches. *Nature Medicine*.

[10] Pascoe J. R., Ferraro G. L., Cannon J. H., Arthur R. M., Wheat J. D. (1981). Exercise induced pulmonary hemorrhage in racing thoroughbreds: a preliminary study. *American Journal of Veterinary Research*.

[11] Raphel C. F., Soma L. R. (1982). Exercise-induced pulmonary hemorrhage in Thoroughbreds after racing and breezing. *American Journal of Veterinary Research*.

[12] Newton J. R., Wood J. L. (2002). Evidence of an association between inflammatory airway disease and EIPH in young Thoroughbreds during training. *Equine Veterinary Journal. Supplement*.

[13] Wood J. L. N., Newton J. R., Chanter N., Mumford J. A. (2005). Association between respiratory disease and bacterial and viral infections in British racehorses. *Journal of Clinical Microbiology*.

[14] Deegen E. Das chronisch lungenkranke Pferd und sein Einsatz im Sport.

[15] Viel L. (1980). *Structural-functional correlations of the lung in the normal light horse [M.S. thesis]*.

[16] Bjermer L. (2007). Time for a paradigm shift in asthma treatment: from relieving bronchospasm to controlling systemic inflammation. *Journal of Allergy and Clinical Immunology*.

[17] Rosenberg S. R., Kalhan R. (2012). Biomarkers in chronic obstructive pulmonary disease. *Translational Research*.

[18] Koutsokera A., Stolz D., Loukides S., Kostikas K. (2012). Systemic biomarkers in exacerbations of COPD: the evolving clinical challenge. *Chest*.

[19] Derksen F. J., Scott J. S., Miller D. C., Slocombe R. F., Robinson N. E. (1985). Bronchoalveolar lavage in ponies with recurrent airway-obstruction (heaves). *American Review of Respiratory Disease*.

[20] Schusser G. F., Wiegand M., Ruhland A. (1999). Technique and cell differential in bronchoalveolar lavage of horses with COPD. *Der Praktische Tierarzt*.

[21] Tremblay G. M., Ferland C., Lapointe J. M., Vrins A., Lavoie J. P., Cormier Y. (1993). Effect of stabling on bronchoalveolar cells obtained from normal and COPD horses. *Equine Veterinary Journal*.

[22] Fairbairn S. M., Page C. P., Lees P., Cunningham F. M. (1993). Early neutrophil but not eosinophil or platelet recruitment to the lungs of allergic horses following antigen exposure. *Clinical and Experimental Allergy*.

[23] Brazil T. J., Dagleish M. P., McGorum B. C., Dixon P. M., Haslett C., Chilvers E. R. (2005). Kinetics of pulmonary neutrophil recruitment and clearance in a natural and spontaneously resolving model of airway inflammation. *Clinical and Experimental Allergy*.

[24] Franchini M., Gilli U., Akens M. K., Fellenberg R. V., Bracher V. (1998). The role of neutrophil chemotactic cytokines in the pathogenesis of equine chronic obstructive pulmonary disease (COPD). *Veterinary Immunology and Immunopathology*.

[25] Lopuhaä C. E., Out T. A., Jansen H. M., Aalberse R. C., Van der Zee J. S. (2002). Allergen-induced bronchial inflammation in house dust mite-allergic patients with or without asthma. *Clinical and Experimental Allergy*.

[26] Norzila M. Z., Fakes K., Henry R. L., Simpson J., Gibson P. G. (2000). Interleukin-8 secretion and neutrophil recruitment accompanies induced sputum eosinophil activation in children with acute asthma. *American Journal of Respiratory and Critical Care Medicine*.

[27] Ordoñez C. L., Shaughnessy T. E., Matthay M. A., Fahy J. V. (2000). Increased neutrophil numbers and IL-8 levels in airway secretions in acute severe asthma: clinical and biologic significance. *American Journal of Respiratory and Critical Care Medicine*.

[28] Robinson N. E. (2001). International workshop on equine chronic airway disease Michigan State University 16–18 June 2000. *Equine Veterinary Journal*.

[29] Fogarty U., Buckley T. (1991). Bronchoalveolar lavage findings in horses with exercise intolerance. *Equine veterinary journal*.

[30] Couëtil L. L., Denicola D. B. (1999). Blood gas, plasma lactate and bronchoalveolar lavage cytology analyses in racehorses with respiratory disease. *Equine Veterinary Journal*.

[31] Moore B. R., Krakowka S., Robertson J. T., Cummins J. M. (1995). Cytologic evaluation of bronchoalveolar lavage fluid obtained from standardbred racehorses with inflammatory airway disease. *American Journal of Veterinary Research*.

[32] Holcombe S. J., Jackson C., Gerber V. (2001). Stabling is associated with airway inflammation in young Arabian horses. *Equine Veterinary Journal*.

[33] Couëtil L. L., Rosenthal F. S., DeNicola D. B., Chilcoat C. D. (2001). Clinical signs, evaluation of bronchoalveolar lavage fluid, and assessment of pulmonary function in horses with inflammatory respiratory disease. *American Journal of Veterinary Research*.

[34] Sánchez A., Couëtil L. L., Ward M. P., Clark S. P. (2005). Effect of airway disease on blood gas exchange in racehorses. *Journal of Veterinary Internal Medicine*.

[35] Hare J. E., Viel L., O'Byrne P. M., Conlon P. D. (1994). Effect of sodium cromoglycate on light racehorses with elevated metachromatic cell numbers on bronchoalveolar lavage and reduced exercise tolerance. *Journal of Veterinary Pharmacology and Therapeutics*.

[36] Hoffman A. M., Mazan M. R., Ellenberg S. (1998). Association between bronchoalveolar lavage cytologic features and airway reactivity in horses with a history of exercise intolerance. *American Journal of Veterinary Research*.

[37] Hare J. E., Viel L. (1998). Pulmonary eosinophilia associated with increased airway responsiveness in young racing horses. *Journal of Veterinary Internal Medicine*.

[38] Ainsworth D. M., Grünig G., Matychak M. B. (2003). Recurrent airway obstruction (RAO) in horses is characterized by IFN-*γ* and IL-8 production in bronchoalveolar lavage cells. *Veterinary Immunology and Immunopathology*.

[39] Ainsworth D. M., Appleton J. A., Antczak D. F., Santiago M. A., Aviza G. (2002). IgG antibody responses to an inhaled antigen in horses with 'heaves' (recurrent airway obstruction). *Veterinary Immunology and Immunopathology*.

[40] Gerber V., Robinson N. E., Venta P. J., Rawson J., Jefcoat A. M., Hotchkiss J. A. (2003). Mucin genes in horse airways: MUC5AC, but not MUC2, may play a role in recurrent airway obstruction. *Equine Veterinary Journal*.

[41] Lindberg Å., Näsman-Glaser B., Lindgren J. Å., Robinson N. E. (2002). Evaluation of leukotriene biosynthetic capacity in lung tissues from horses with recurrent airway obstruction. *American Journal of Veterinary Research*.

[42] Jackson C. A., Berney C., Jefcoat A. M., Robinson N. E. (2000). Environment and prednisone interactions in the treatment of recurrent airway obstruction (heaves). *Equine Veterinary Journal*.

[43] Robinson N. E., Jackson C. A., Peroni D. (2000). Why is oral prednisone ineffective for treatment of heaves?. *American Association of Equine Practitioners*.

[44] Robinson N. E., Olszewski M. A., Boehler D. (2000). Relationship between clinical signs and lung function in horses with recurrent airway obstruction (heaves) during a bronchodilator trial. *Equine Veterinary Journal*.

[45] Rush B. R., Raub E. S., Thomsen M. M., Davis E. G., Matson C. J., Hakala J. E. (2000). Pulmonary function and adrenal gland suppression with incremental doses of aerosolized beclomethasone dipropionate in horses with recurrent airway obstruction. *Journal of the American Veterinary Medical Association*.

[46] Henrikson S. L., Rush B. R. (2001). Efficacy of salmeterol xinafoate in horses with recurrent airway obstruction. *Journal of the American Veterinary Medical Association*.

[47] Lavoie J.-P., Léguillette R., Pasloske K. (2002). Comparison of effects of dexamethasone and the leukotriene D4 receptor antagonist L-708,738 on lung function and airway cytologic findings in horses with recurrent airway obstruction. *American Journal of Veterinary Research*.

[48] Léguillette R., Désévaux C., Lavoie J.-P. (2002). Effects of pentoxifylline on pulmonary function and results of cytologic examination of bronchoalveolar lavage fluid in horses with recurrent airway obstruction. *American Journal of Veterinary Research*.

[49] Robinson N. E., Jackson C., Jefcoat A., Berney C., Peroni D., Derksen F. J. (2002). Efficacy of three corticosteroids for the treatment of heaves. *Equine Veterinary Journal*.

[50] Robinson N. E., Jefcoat A. M., Gerber V. (2002). Mucus and inflammation in equine heaves. *Pferdeheilkunde*.

[51] Picandet V., Léguillette R., Lavoie J.-P. (2003). Comparison of efficacy and tolerability of isoflupredone and dexamethasone in the treatment of horses affected with recurrent airway obstruction (‘heaves’). *Equine Veterinary Journal*.

[52] Mazan M. R., Hoffman A. M., Kuehn H., Deveney E. F. (2003). Effect of aerosolized albuterol sulfate on resting energy expenditure determined by use of open-flow indirect calorimetry in horses with recurrent airway obstruction. *American Journal of Veterinary Research*.

[53] Rickards K. J., Page C. P., Cunningham F. M. (2004). Allergen challenge alters lymphocyte phosphodiesterase activity in horses with heaves. *Pulmonary Pharmacology and Therapeutics*.

[54] Lavoie J.-P., Maghni K., Desnoyers M., Taha R., Martin J. G., Hamid Q. A. (2001). Neutrophilic airway inflammation in horses with heaves is characterized by a Th2-type cytokine profile. *American Journal of Respiratory and Critical Care Medicine*.

[55] Beadle R. E., Horohov D. W., Gaunt S. D. (2002). Interleukin-4 and interferon-gamma gene expression in summer pasture-associated obstructive pulmonary disease affected horses. *Equine Veterinary Journal*.

[56] Cordeau M.-E., Joubert P., Dewachi O., Hamid Q., Lavoie J.-P. (2004). IL-4, IL-5 and IFN-*γ* mRNA expression in pulmonary lymphocytes in equine heaves. *Veterinary Immunology and Immunopathology*.

[57] Gerber V., Swinburne J. E., Blott S. C. (2008). Genetics of recurrent airway obstruction (RAO). *Deutsche Tierarztliche Wochenschrift*.

[58] Kleiber C., McGorum B. C., Horohov D. W., Pirie R. S., Zurbriggen A., Straub R. (2005). Cytokine profiles of peripheral blood and airway CD4 and CD8 T lymphocytes in horses with recurrent airway obstruction. *Veterinary Immunology and Immunopathology*.

[59] Klier J., Fuchs S., May A. (2012). A nebulized gelatin nanoparticle-based CpG formulation is effective in immunotherapy of allergic horses. *Pharmaceutical Research*.

[60] Franchini M., Gill U., Von Fellenberg R., Bracher V. D. (2000). Interleukin-8 concentration and neutrophil chemotactic activity in bronchoalveolar lavage fluid of horses with chronic obstructive pulmonary disease following exposure to hay. *American Journal of Veterinary Research*.

[61] Debrue M., Hamilton E., Joubert P., Lajoie-Kadoch S., Lavoie J.-P. (2005). Chronic exacerbation of equine heaves is associated with an increased expression of interleukin-17 mRNA in bronchoalveolar lavage cells. *Veterinary Immunology and Immunopathology*.

[62] Ainsworth D. M., Wagner B., Erb H. N. (2007). Effects of in vitro exposure to hay dust on expression of interleukin-17, -23,-8, and -1*β* and chemokine (C-X-C motif) ligand 2 by pulmonary mononuclear cells isolated from horses chronically affected with recurrent airway disease. *American Journal of Veterinary Research*.

[63] Art T., Franck T., Lekeux P. (2006). Myeloperoxidase concentration in bronchoalveolar lavage fluid from healthy horses and those with recurrent airway obstruction. *Canadian Journal of Veterinary Research*.

[64] Fey K. (2004). *Der klinische Nutzen Zytologischer Untersuchungen von Broncho-Alveolärer Lavageflüssigkeit bei der Differenzierung Chronischer Bronchitiden des Pferdes*.

[65] Deaton C. M., Marlin D. J., Deaton L. (2006). Comparison of the antioxidant status in tracheal and bronchoalveolar epithelial lining fluids in recurrent airway obstruction. *Equine Veterinary Journal*.

[66] Deaton C. M. (2006). The role of oxidative stress in an equine model of human asthma. *Redox Report*.

[67] Deaton C. M., Marlin D. J., Smith N. C. (2005). Antioxidant and inflammatory responses of healthy horses and horses affected by recurrent airway obstruction to inhaled ozone. *Equine Veterinary Journal*.

[68] Deaton C. M., Marlin D. J., Smith N. C. (2005). Effect of acute airway inflammation on the pulmonary antioxidant status. *Experimental Lung Research*.

[69] Deaton C. M., Marlin D. J., Smith N. C. (2004). Pulmonary epithelial lining fluid and plasma ascorbic acid concentrations in horses affected by recurrent airway obstruction. *American Journal of Veterinary Research*.

[70] Wood L. G., Baines K. J., Fu J., Scott H. A., Gibson P. G. (2012). The neutrophilic inflammatory phenotype is associated with systemic inflammation in asthma. *Chest*.

[71] Miskovic M., Couëtil L. L., Thompson C. A. (2007). Lung function and airway cytologic profiles in horses with recurrent airway obstruction maintained in low-dust environments. *Journal of Veterinary Internal Medicine*.

[72] Sandersen C., Bureau F., Turlej R. (2001). p65 Homodimer activity in distal airway cells determines lung dysfunction in equine heaves. *Veterinary Immunology and Immunopathology*.

[73] Lavoie-Lamoureux A., Moran K., Beauchamp G. (2010). IL-4 activates equine neutrophils and induces a mixed inflammatory cytokine expression profile with enhanced neutrophil chemotactic mediator release ex vivo. *American Journal of Physiology - Lung Cellular and Molecular Physiology*.

[74] Rickards K. J., Page C. P., Lees P., Gettinby G., Cunningham F. M. (2003). In vitro and ex vivo effects of the phosphodiesterase 4 inhibitor, rolipram, on thromboxane production in equine blood. *Journal of Veterinary Pharmacology and Therapeutics*.

[75] Marr K. A., Lees P., Cunningham F. M. (2002). Antigen challenge increases adherence of circulating neutrophils in horses with chronic obstructive pulmonary disease. *Equine Veterinary Journal*.

[76] Kirschvink N., Smith N., Fiévez L. (2002). Effect of chronic airway inflammation and exercise on pulmonary and systemic antioxidant status of healthy and heaves-affected horses. *Equine Veterinary Journal*.

[77] Gray P. R., Derksen F. J., Robinson N. E., Carpenter-Deyo L. J., Johnson H. G., Roth R. A. (1989). The role of cyclooxygenase products in the acute airway obstruction and airway hyperreactivity of ponies with heaves. *American Review of Respiratory Disease*.

[78] Kasayama S., Tanemura M., Koga M., Fujita K., Yamamoto H., Miyatake A. (2009). Asthma is an independent risk for elevation of plasma C-reactive protein levels. *Clinica Chimica Acta*.

[79] Koh Y. Y., Kim Y. W., Park J. D., Oh J. W. (1996). A comparison of serum haptoglobin levels between acute exacerbation and clinical remission in asthma. *Clinical and Experimental Allergy*.

[80] Wu T.-L., Chang P.-Y., Tsao K.-C., Sun C.-F., Wu L. L., Wu J. T. (2007). A panel of multiple markers associated with chronic systemic inflammation and the risk of atherogenesis is detectable in asthma and chronic obstructive pulmonary disease. *Journal of Clinical Laboratory Analysis*.

[81] Schanen J. G., Iribarren C., Shahar E. (2005). Asthma and incident cardiovascular disease: The Atherosclerosis Risk In Communities Study. *Thorax*.

[82] Song Y., Klevak A., Manson J. E., Buring J. E., Liu S. (2010). Asthma, chronic obstructive pulmonary disease, and type 2 diabetes in the Women's Health Study. *Diabetes Research and Clinical Practice*.

[83] Sin D. D., Anthonisen N. R., Soriano J. B., Agusti A. G. (2006). Mortality in COPD: role of comorbidities. *European Respiratory Journal*.

[84] Yende S., Waterer G. W., Tolley E. A. (2006). Inflammatory markers are associated with ventilatory limitation and muscle dysfunction in obstructive lung disease in well functioning elderly subjects. *Thorax*.

[85] Lavoie-Lamoureux A., Leclere M., Lemos K., Wagner B., Lavoie J.-P. (2012). Markers of systemic inflammation in horses with heaves. *Journal of Veterinary Internal Medicine*.

[86] Lavoie-Lamoureux A., Beauchamp G., Quessy S., Martin J. G., Lavoie J.-P. (2012). Systemic inflammation and priming of peripheral blood leukocytes persist during clinical remission in horses with heaves. *Veterinary Immunology and Immunopathology*.

[87] Niedźwiedź A., Nicpoń J., Borowicz H., LŁoś P., Zawadzki M., Januszewska L. (2011). Evaluation of selected antioxidant enzymes in the blood of horses with recurrent airways obstruction. *Medycyna Weterynaryjna*.

[88] Müller B., White J. C., Nylén E. S., Snider R. H., Becker K. L., Habener J. F. (2001). Ubiquitous expression of the calcitonin-I gene in multiple tissues in response to sepsis. *The Journal of Clinical Endocrinology & Metabolism*.

[89] Pereira J. M., Teixeira-Pinto A., Basílio C., Sousa-Dias C., Mergulhão P., Paiva J. A. (2013). Can we predict pneumococcal bacteremia in patients with severe community-acquired pneumonia?. *Journal of Critical Care*.

[90] Julián-Jiménez A., Timón Zapata J., Laserna Mendieta E. J. (2014). Diagnostic and prognostic power of biomarkers to improve the management of community acquired pneumonia in the emergency department. *Enfermedades Infecciosas y Microbiología Clínica*.

[91] Berg P., Lindhardt B. Ø. (2012). The role of procalcitonin in adult patients with community-acquired pneumonia—a systematic review. *Danish Medical Journal*.

[92] Niu W., Wan Y.-G., Li M.-Y., Wu Z.-X., Zhang L.-G., Wang J.-X. (2013). The diagnostic value of serum procalcitonin, IL-10 and C-reactive protein in community acquired pneumonia and tuberculosis. *European Review for Medical and Pharmacological Sciences*.

[93] Tang J., Long W., Yan L. (2013). Procalcitonin guided antibiotic therapy of acute exacerbations of asthma: a randomized controlled trial. *BMC Infectious Diseases*.

[94] Walsh E. E., Swinburne A. J., Becker K. L. (2013). Can serum procalcitonin levels help interpret indeterminate chest radiographs in patients hospitalized with acute respiratory illness?. *Journal of Hospital Medicine*.

[95] Brightling C. E. (2013). Biomarkers that predict and guide therapy for exacerbations of chronic obstructive pulmonary disease. *Annals of the American Thoracic Society*.

[96] Zhao Y.-F., Jiang Y.-P., Zhou L.-F., Wu X.-L. (2014). The value of assessment tests in patients with acute exacerbation of chronic obstructive pulmonary disease. *American Journal of the Medical Sciences*.

[97] Rieger M., Kochleus C., Teschner D. (2014). A new ELISA for the quantification of equine procalcitonin in plasma as potential inflammation biomarker in horses. *Analytical and Bioanalytical Chemistry*.

[98] Teschner D., Rieger M., Koopmann C., Gehlen H. (2015). Procalcitonin in horses with an acute colic. *Pferdeheilkunde*.

[99] Bonelli F., Meucci V., Divers T. J. (2015). Plasma procalcitonin concentration in healthy horses and horses affected by systemic inflammatory response syndrome. *Journal of Veterinary Internal Medicine*.

[100] Barton A., Shety T., Pelli A. Evaluation of inflammatory markers and treatment success in inflammatory airway disease under the course of inhalation therapy and environmental dust reduction.

[101] Ainsworth D. M. Review of recurrent airway obstruction (RAO, Heaves): diagnosis and treatment options.

[102] Lavoie J. P., Martin B. J., Ainsworth M., Viel R. Lung remodeling in the horse with heaves. http://www.ivis.org/.

[103] Kaup F. J., Drommer W., Deegen E. (1990). Ultrastructural findings in horses with chronic obstructive pulmonary disease (COPD) I: alterations of the larger conducting airways. *Equine Veterinary Journal*.

[104] Kaup F. J., Drommer W., Damsch S., Deegen E. (1990). Ultrastructural findings in horses with chronic obstructive pulmonary disease (COPD) II: pathomorphological changes of the terminal airways and the alveolar region. *Equine Veterinary Journal*.

[105] Seeger W., Stohr G., Wolf H. R. D., Neuhof H. (1985). Alteration of surfactant function due to protein leakage: special interaction with fibrin monomer. *Journal of Applied Physiology*.

[106] Günther A., Mosavi P., Heinemann S. (2000). Alveolar fibrin formation caused by enhanced procoagulant and depressed fibrinolytic capacities in severe pneumonia: comparison with the acute respiratory distress syndrome. *American Journal of Respiratory and Critical Care Medicine*.

[107] Akinnusi M. E., El Solh A. A. (2007). The role of coagulation in pulmonary pathology. *Inflammation and Allergy—Drug Targets*.

[108] Nakstad B., Lyberg T., Skjønsberg O. H., Boye N. P. (1990). Local activation of the coagulation and fibrinolysis systems in lung disease. *Thrombosis Research*.

[109] Idell S. (2003). Coagulation, fibrinolysis, and fibrin deposition in acute lung injury. *Critical Care Medicine*.

[110] Ciano P. S., Colvin R. B., Dvorak A. M., McDonagh J. (1986). Macrophage migration in fibrin gel matrices. *Laboratory Investigation*.

[111] Leavell K. J., Peterson M. W., Gross T. J. (1996). The role of fibrin degradation products in neutrophil recruitment to the lung. *American Journal of Respiratory Cell and Molecular Biology*.

[112] Grinnell F., Feld M., Minter D. (1980). Fibroblast adhesion to fibrinogen and fibrin substrata: requirement for cold-insoluble globulin (plasma fibronectin). *Cell*.

[113] Gray A. J., Bishop J. E., Reeves J. T., Mecham R. P., Laurent G. J. (1995). Partially degraded fibrin(ogen) stimulates fibroblast proliferation in vitro. *American Journal of Respiratory Cell and Molecular Biology*.

[114] Sitrin R. G., Pan P. M., Srikanth S., Todd R. F. (1998). Fibrinogen activates NF-*κ*B transcription factors in mononuclear phagocytes. *The Journal of Immunology*.

[115] Seeger W., Elssner A., Günther A., Krämer H.-J., Kalinowski H. O. (1993). Lung surfactant phospholipids associate with polymerizing fibrin: loss of surface activity. *American journal of respiratory cell and molecular biology*.

[116] Ware L. B., Bastarache J. A., Wang L. (2005). Coagulation and fibrinolysis in human acute lung injury—new therapeutic targets?. *Keio Journal of Medicine*.

[117] Schuliga M., Royce S. G., Langenbach S. (2016). The coagulant factor Xa induces protease-activated receptor-1 and annexin A2-dependent airway smooth muscle cytokine production and cell proliferation. *American Journal of Respiratory Cell and Molecular Biology*.

[118] Winder N. C., Grünig G., Hermann M., von Fellenberg R. (1990). Fibrin/fibrinogen in lungs and respiratory secretions of horses with chronic pulmonary disease. *American Journal of Veterinary Research*.

[119] Delgado M. A., Monreal L., Armengou L., Ríos J., Segura D. (2009). Peritoneal D-dimer concentration for assessing peritoneal fibrinolytic activity in horses with colic. *Journal of Veterinary Internal Medicine*.

[120] Monreal L., Anglés A., Espada Y. (2000). Hypercoagulation and hypofibrinolysis in horses with colic and DIC. *Equine Veterinary Journal*.

[121] Ribera T., Monreal L., Armengou L., Ríos J., Prades M. (2011). Synovial fluid D-dimer concentration in foals with septic joint disease. *Journal of Veterinary Internal Medicine*.

[122] Clutterbuck A. L., Harris P., Allaway D., Mobasheri A. (2010). Matrix metalloproteinases in inflammatory pathologies of the horse. *Veterinary Journal*.

[123] Dunsmore S. E., Rannels D. E. (1996). Extracellular matrix biology in the lung. *American Journal of Physiology—Lung Cellular and Molecular Physiology*.

[124] Shapiro S. D., Endicott S. K., Province M. A., Pierce J. A., Campbell E. J. (1991). Marked longevity of human lung parenchymal elastic fibers deduced from prevalence of D-aspartate and nuclear weapons-related radiocarbon. *The Journal of Clinical Investigation*.

[125] Foronjy R. F., Okada Y., Cole R., D'Armiento J. (2003). Progressive adult-onset emphysema in transgenic mice expressing human MMP-1 in the lung. *American Journal of Physiology—Lung Cellular and Molecular Physiology*.

[126] Shapiro S. D., Campbell E. J., Welgus H. G., Senior R. M. (1991). Elastin degradation by mononuclear phagocytes. *Annals of the New York Academy of Sciences*.

[127] Gross J., Lapiere C. M. (1962). Collagenolytic activity in amphibian tissues: a tissue culture assay. *Proceedings of the National Academy of Sciences of the United States of America*.

[128] Ratjen F., Hartog C.-M., Paul K., Wermelt J., Braun J. (2002). Matrix metalloproteases in BAL fluid of patients with cystic fibrosis and their modulation by treatment with dornase alpha. *Thorax*.

[129] Krizkova S., Zitka O., Masarik M. (2011). Clinical importance of matrix metalloproteinases. *Bratislava Medical Journal*.

[130] Naito Y., Yoshikawa T. (2005). Role of matrix metalloproteinases in inflammatory bowel disease. *Molecular Aspects of Medicine*.

[131] Brinckerhoff C. E., Matrisian L. M. (2002). Matrix metalloproteinases: a tail of a frog that became a prince. *Nature Reviews Molecular Cell Biology*.

[132] Parks W. C., Wilson C. L., López-Boado Y. S. (2004). Matrix metalloproteinases as modulators of inflammation and innate immunity. *Nature Reviews Immunology*.

[133] Atkinson J. J., Lutey B. A., Suzuki Y. (2011). The role of matrix metalloproteinase-9 in cigarette smoke-induced emphysema. *American Journal of Respiratory and Critical Care Medicine*.

[134] Russell R. E. K., Culpitt S. V., DeMatos C. (2002). Release and activity of matrix metalloproteinase-9 and tissue inhibitor of metalloproteinase-1 by alveolar macrophages from patients with chronic obstructive pulmonary disease. *American Journal of Respiratory Cell and Molecular Biology*.

[135] Finlay G. A., Russell K. J., McMahon K. J. (1997). Elevated levels of matrix metalloproteinases in bronchoalveolar lavage fluid of emphysematous patients. *Thorax*.

[136] Finlay G. A., O'Driscoll L. R., Russell K. J. (1997). Matrix metalloproteinase expression and production by alveolar macrophages in emphysema. *American Journal of Respiratory and Critical Care Medicine*.

[137] Koivunen A.-L., Maisi P., Konttinen Y. T., Sandholm M. (1997). Gelatinolytic activity in tracheal aspirates of horses with chronic obstructive pulmonary disease. *Acta Veterinaria Scandinavica*.

[138] Barton A. K., Shety T., Bondzio A., Einspanier R., Gehlen H. (2015). Metalloproteinases and their tissue inhibitors in comparison between different chronic pneumopathies in the horse. *Mediators of Inflammation*.

[139] Nevalainen M., Raulo S. M., Brazil T. J. (2002). Inhalation of organic dusts and lipopolysaccharide increases gelatinolytic matrix metalloproteinases (MMPs) in the lungs of heaves horses. *Equine Veterinary Journal*.

[140] Simonen-Jokinen T., Pirie R. S., McGorum B. C., Maisi P. (2005). Effect of composition and different fractions of hay dust suspension on inflammation in lungs of heaves-affected horses: MMP-9 and MMP-2 as indicators of tissue destruction. *Equine Veterinary Journal*.

[141] Simonen-Jokinen T., Pirie R. S., McGorum B., Maisi P. (2005). Dose responses to inhalation of endotoxin, hay dust suspension and Aspergillus fumigatus extract in horses as measured by levels and activation of matrix metalloproteinase-9. *Equine Veterinary Journal*.

[142] Barton A., Shety T., Bondzio A. (2013). *Changes in MMP-2, MMP-9 and IL-8 in BALF of RAO Horses before and after Therapy*.

[143] Koivunen A.-L., Maisi P., Konttinen Y. T., Prikk K., Sandholm M. (1997). Collagenolytic activity and its sensitivity to doxycycline inhibition in tracheal aspirates of horses with chronic obstructive pulmonary disease. *Acta Veterinaria Scandinavica*.

[144] Raulo S. M., Sorsa T., Tervahartiala T. (2001). MMP-9 as a marker of inflammation in tracheal epithelial lining fluid (TELF) and in bronchoalveolar fluid (BALF) of COPD horses. *Equine Veterinary Journal*.

[145] Raulo S. M., Sorsa T. A., Kiili M. T., Maisi P. S. (2001). Evaluation of collagenase activity, matrix metalloproteinase-8, and matrix metalloproteinase-13 in horses with chronic obstructive pulmonary disease. *American Journal of Veterinary Research*.

[146] Elkington P. T. G., Friedland J. S. (2006). Matrix metalloproteinases in destructive pulmonary pathology. *Thorax*.

[147] Bourboulia D., Stetler-Stevenson W. G. (2010). Matrix metalloproteinases (MMPs) and tissue inhibitors of metalloproteinases (TIMPs): positive and negative regulators in tumor cell adhesion. *Seminars in Cancer Biology*.

[148] Brew K., Nagase H. (2010). The tissue inhibitors of metalloproteinases (TIMPs): an ancient family with structural and functional diversity. *Biochimica et Biophysica Acta—Molecular Cell Research*.

[149] Ugarte-Gil C. A., Elkington P., Gilman R. H. (2013). Induced sputum MMP-1, -3 & -8 concentrations during treatment of tuberculosis. *PLoS ONE*.

[150] D'Armiento J. M., Goldklang M. P., Hardigan A. A. (2013). Increased matrix metalloproteinase (MMPs) levels do not predict disease severity or progression in emphysema. *PLoS ONE*.

[151] Oka S., Furukawa H., Shimada K. (2013). Serum biomarker analysis of collagen disease patients with acute-onset diffuse interstitial lung disease. *BMC Immunology*.

[152] Broadstone R. V., LeBlanc P. H., Derksen F. J., Robinson N. E. (1991). In vitro responses of airway smooth muscle from horses with recurrent airway obstruction. *Pulmonary Pharmacology*.

[153] Leclere M., Lavoie-Lamoureux A., Gélinas-Lymburner É., David F., Martin J. G., Lavoie J.-P. (2011). Effect of antigenic exposure on airway smooth muscle remodeling in an equine model of chronic asthma. *American Journal of Respiratory Cell and Molecular Biology*.

[154] Leclere M., Lavoie-Lamoureux A., Joubert P. (2012). Corticosteroids and antigen avoidance decrease airway smooth muscle mass in an equine asthma model. *American Journal of Respiratory Cell and Molecular Biology*.

[155] Leclere M., Lavoie-Lamoureux A., Joubert P. (2012). Corticosteroids and antigen avoidance decrease airway smooth muscle mass in an equine asthma model. *American Journal of Respiratory Cell and Molecular Biology*.

[156] Yeganeh B., Xia C., Movassagh H. (2013). Emerging mediators of airway smooth muscle dysfunction in asthma. *Pulmonary Pharmacology and Therapeutics*.

[157] Jarai G., Sukkar M., Garrett S. (2004). Effects of interleukin-1*β*, interleukin-13 and transforming growth factor-*β* on gene expression in human airway smooth muscle using gene microarrays. *European Journal of Pharmacology*.

[158] Issa R., Xie S., Lee K. Y. (2006). GRO-*α* regulation in airway smooth muscle by IL-1*β* and TNF-*α*: role of NF-*κ*B and MAP kinases. *American Journal of Physiology. Lung Cellular and Molecular Physiology*.

[159] Sergejeva S., Ivanov S., Lötvall J., Lindén A. (2005). Interleukin-17 as a recruitment and survival factor for airway macrophages in allergic airway inflammation. *American Journal of Respiratory Cell and Molecular Biology*.

[160] Roussel L., Houle F., Chan C. (2010). IL-17 promotes p38 MAPK-dependent endothelial activation enhancing neutrophil recruitment to sites of inflammation. *The Journal of Immunology*.

[161] Prause O., Bozinovski S., Anderson G. P., Lindén A. (2004). Increased matrix metalloproteinase-9 concentration and activity after stimulation with interleukin-17 in mouse airways. *Thorax*.

[162] Lindén A. (2001). Role of interleukin-17 and the neutrophil in asthma. *International Archives of Allergy and Immunology*.

[163] Ainsworth D. M., Wagner B., Franchini M., Grünig G., Erb H. N., Tan J.-Y. (2006). Time-dependent alterations in gene expression of interleukin-8 in the bronchial epithelium of horses with recurrent airway obstruction. *American Journal of Veterinary Research*.

[164] Padoan E., Ferraresso S., Pegolo S., Castagnaro M., Barnini C., Bargelloni L. (2013). Real time RT-PCR analysis of inflammatory mediator expression in recurrent airway obstruction-affected horses. *Veterinary Immunology and Immunopathology*.

[165] Linden A., Dahlen B. (2014). Interleukin-17 cytokine signalling in patients withasthma. *European Respiratory Journal*.

[166] Kolodkin A. L., Matthes D. J., Goodman C. S. (1993). The semaphorin genes encode a family of transmembrane and secreted growth cone guidance molecules. *Cell*.

[167] Al-Muhsen S., Johnson J. R., Hamid Q. (2011). Remodeling in asthma. *Journal of Allergy and Clinical Immunology*.

[168] Gan Y., Reilkoff R., Peng X. (2011). Role of semaphorin 7a signaling in transforming growth factor *β*1-induced lung fibrosis and scleroderma-related interstitial lung disease. *Arthritis and Rheumatism*.

